# Can a warm and supportive adult protect against mental health problems amongst children with experience of adversity? A twin‐differences study

**DOI:** 10.1111/jcpp.14070

**Published:** 2024-11-12

**Authors:** Sarah E. Stock, Rebecca E. Lacey, Louise Arseneault, Avshalom Caspi, Eloise Crush, Andrea Danese, Rachel M. Latham, Terrie E. Moffitt, Joanne B. Newbury, Jonathan D. Schaefer, Helen L. Fisher, Jessie R. Baldwin

**Affiliations:** ^1^ Research Department of Epidemiology and Public Health University College London London UK; ^2^ School of Health and Medical Sciences, City St George's University of London London UK; ^3^ Social Genetic and Developmental Psychiatry Centre, Institute of Psychiatry, Psychology and Neuroscience King's College London London UK; ^4^ ESRC Centre for Society and Mental Health King's College London London UK; ^5^ Department of Psychology and Neuroscience Duke University Durham NC USA; ^6^ Duke University Population Research Institute Duke University Durham NC USA; ^7^ Department of Psychiatry and Behavioral Sciences Duke University Durham NC USA; ^8^ PROMENTA, Department of Psychology University of Oslo Oslo Norway; ^9^ Department of Child and Adolescent Psychiatry, Institute of Psychiatry, Psychology and Neuroscience King's College London London UK; ^10^ National and Specialist CAMHS Trauma, Anxiety, and Depression Clinic South London and Maudsley NHS Foundation Trust London UK; ^11^ Population Health Sciences: Bristol Medical School University of Bristol Bristol UK; ^12^ Institute of Child Development University of Minnesota Minneapolis MN USA; ^13^ Division of Psychology and Language Sciences, Department of Clinical, Educational and Health Psychology University College London London UK

**Keywords:** Protective factors, ACEs, twin differences, adult social support, maternal warmth, psychopathology, resilience

## Abstract

**Background:**

Adverse childhood experiences (ACEs) are associated with mental health problems, but many children who experience ACEs do not develop such difficulties. A warm and supportive adult presence in childhood is associated with a lower likelihood of developing mental health problems after exposure to ACEs. However, it is unclear whether this association is causal, as previous research has not accounted for genetic and environmental confounding.

**Methods:**

We used the twin‐difference design to strengthen causal inference about whether a warm and supportive adult presence protects children exposed to ACEs from mental health problems. Participants were from the Environmental Risk (E‐Risk) Longitudinal Twin Study, a UK population‐representative birth cohort of 2,232 same‐sex twins. ACEs were measured prospectively from ages 5 to 12. Maternal warmth was assessed at ages 5 and 10 through maternal speech samples. Adult support was assessed through child reports at age 12. Mental health problems were assessed through interviews at age 12 with parents and teachers and participants at age 18.

**Results:**

Among children exposed to ACEs, those who experienced greater maternal warmth and adult support had lower levels of mental health problems at ages 12 and 18. In monozygotic twin‐difference analyses, the protective effects of maternal warmth and adult support on mental health were attenuated by 70% for maternal warmth and 81% for adult support, compared to phenotypic analyses. Twins who experienced greater maternal warmth and adult support had minimal or no difference in mental health compared to their co‐twins, concordant for ACE exposure.

**Conclusions:**

The apparent protective effect of a warm, supportive adult against mental health problems following ACEs is largely explained by genetic and environmental confounding. This suggests that interventions which boost maternal warmth and adult support should be supplemented by components addressing wider family environments and heritable vulnerabilities in children exposed to adversity, to improve mental health.

## Introduction

Adverse childhood experiences (ACEs), such as abuse, neglect and dysfunctional family environments, are associated with adolescent mental health problems (Gondek, Patalay, & Lacey, 2021). However, individual differences exist in childrens' responses to ACEs, and many exposed children do not develop mental health problems (Baldwin et al., 2021). Such resilience to childhood adversity may in part be explained by support from a warm and caring adult, which has been associated with positive mental health among children exposed to ACEs (Crouch, Radcliff, Strompolis, & Srivastav, 2019; Fritz, de Graaff, Caisley, van Harmelen, & Wilkinson, 2018; Sciaraffa, Zeanah, & Zeanah, 2018). However, it is not clear if a warm and supportive adult presence has a casual protective effect in the context of ACEs, as most existing research has not accounted for genetic and environmental confounding.

Genetic confounding might arise if genetic influences on mental health problems affect a child's exposure to a warm and supportive adult, via gene–environment correlation (rGE) (Jaffee & Price, 2007). This could occur via three processes. First, a child whose parents have mental health problems may be less likely to receive warmth and support from them, whilst also inheriting genes which predispose them to psychopathology (i.e. passive rGE). Second, a child who is more withdrawn or disruptive may be less likely to elicit a warm, supportive response from adults in their environment (i.e. evocative/reactive rGE). Third, a child who feels anxious or overwhelmed may be more likely to seek out solitary environments where adult warmth and support is not easily accessed (i.e. active rGE). In these ways, it is possible that children with genetic liability to mental health problems have a reduced likelihood of receiving warmth and support from adults. If this is the case, adult warmth and support might be linked to positive mental health outcomes due to genetic confounding, rather than a causal protective effect.

Environmental confounding is also likely to arise because various home environments influence both the likelihood of experiencing adult warmth and support, as well as adolescent mental health. For example, household socioeconomic disadvantage is associated with a lower likelihood of maternal positive parenting (Azad, Blacher, & Marcoulides, 2014) and a greater risk of adolescent mental health difficulties (Ackerman, Brown, & Izard, 2004; Reiss, 2013), relative to advantaged households. Additionally, children who live in an area with neighbourhood social cohesion may have access to support from adults in their community, as well as positive mental health (Latham et al., 2022).

To account for genetic and environmental confounding, the twin‐difference design can be used. The twin‐difference design capitalises on the fact that twins share their genetic material (100% for monozygotic twins and 50% for dizygotic twins), as well as their home environment. Therefore, if a twin who experienced greater levels of adult support has fewer mental health problems than their co‐twin with similar level of ACE exposure, it would suggest that adult support protects against mental health problems in children with ACE exposure, independent of confounding by genetics and the shared family environment. While the twin difference design does not account for confounding from the non‐shared environment (i.e. factors which differ between twins), such factors can be statistically controlled for when they have been measured. Previous twin‐difference studies have suggested causal protective effects of maternal warmth on child behavioural problems, following bullying victimisation (Bowes, Maughan, Caspi, Moffitt, & Arseneault, 2010), and protective effects of social support from friends on psychotic experiences (Crush, Arseneault, Danese, Jaffee, & Fisher, 2020). However, it is not known whether the presence of a warm and supportive adult can protect children exposed to ACEs in the family from developing adolescent mental health problems.

In this study, we utilised the twin‐difference design to test whether the presence of a warm and supportive adult protects against mental health problems in children exposed to adversity. We used prospective longitudinal data from a representative sample of UK twins and examined multiple mental health outcomes in early and late adolescence. To our knowledge, this is the first genetically informed study to examine the protective effects of a warm and supportive adult amongst children exposed to ACEs within the family.

## Methods

### Sample

This study used data from the Environmental Risk (E‐Risk) Longitudinal Twin Study. E‐Risk is a birth cohort of 2,232 same‐sex twins born in England and Wales in 1994–1995 which was drawn from a larger twin birth register (Trouton, Spinath, & Plomin, 2002). The sample was constructed in 1999 and 2000, when the twins were 5 years old, and further details about the sample can be found as reported by Moffitt and the E‐Risk Study Team (2002). E‐Risk is a nationally representative sample, recruited to represent the UK population with new‐born twins in the 1990s, based on both mothers' age and residential location. Families in E‐Risk represent UK households across the spectrum of neighbourhood‐level deprivation (see Figure S1). The initial sample comprised of 56% monozygotic and 44% dizygotic twin pairs, with sex evenly distributed within zygosity (49% male). 90.4% of the sample identified as White, 4.0% as Asian, 1.9% as Black, 0.4% as Mixed race and 3.3% did not report their race.

Data were collected through home visits to E‐Risk families by trained research workers when the children were aged 5, 7, 10, 12 and 18 years. Home visits at ages 5–12 years included assessments with participants as well as their mother (or primary caretaker); the home visit at age 18 included interviews only with the participants. Each twin participant was assessed by a different interviewer. At sample inception, 93% of eligible families with same‐sex twins took part. There was high retention at the follow‐up assessments at ages 7 (98% of the 1,116 E‐Risk Study families), 10 (96%), 12 (96%) and 18 (93%). There were 2,066 E‐Risk participants (47% male) who were assessed at age 18. The average age of the participants at the time of the assessment was 18.4 years (*SD* = 0.36); all interviews were conducted after the 18th birthday. There were no differences between those who did and did not take part at age 18 in terms of socioeconomic status (SES) assessed when the cohort was initially defined (χ^2^ = 0.86, *p* = .65), age‐5 IQ scores (*t* = 0.98, *p* = .33), age‐5 behavioural (*t* = 0.40, *p* = .69) or emotional (*t* = 0.41, *p* = .68) problems or childhood poly‐victimisation (*z* = 0.51, *p* = .61).

The Joint South London and Maudsley and the Institute of Psychiatry Research Ethics Committee approved each phase of the study. Parents gave informed consent and twins gave assent at 5 to 12 years of age and then informed consent at 18 years.

### Measures

#### Adverse childhood experiences

ACEs between birth and age 12 were assessed during 4 home visits when children were 5 to 12 years old, using a combination of caregiver reports and interviewer assessments (Baldwin et al., 2021). The ACEs measured were physical abuse, sexual abuse, emotional abuse and neglect, physical neglect, domestic violence, parental antisocial behaviour, family history of substance abuse, family history of mental health problems and parental separation or divorce. An ACE score was derived that summed the number of different ACEs experienced. Further information is provided in Appendix S1 and Table S1.

#### Mental health outcomes

##### Emotional and behavioural difficulties at age 12

Psychopathology in childhood can be captured by dimensions of emotional and behavioural difficulties (Achenbach, 1991a). We used the Child Behavior Checklist with mothers (Achenbach, 1991b) and the Teachers' Report Form with teachers (Achenbach, 1991c) to assess emotional and behavioural problems at age 12. Mothers were given the instrument as a face‐to‐face interview and teachers responded by mail, with separate questionnaires for each twin. Both informants rated each item as being ‘not true’, ‘somewhat or sometimes true’ or ‘very true or often true’. The reporting period was 6 months before the interview. The Emotional Problems scale is the sum of items in the Withdrawn and Anxious/Depressed scales including items such as ‘cries a lot’, ‘feels too guilty’ and ‘worries’ (Somatic Complaints were not included as this scale was not assessed at age 12). The Behavioural Problems scale is the sum of items from the Delinquency and Aggression scales. Teachers' reports were available for 78% of twins participating at age 12. To account for missingness in teacher reports, mean imputation by baseline characteristics (sex and whether the child had a mother aged 20 years or less at the time of her first child) was used. The internal consistencies of mothers' and teachers' reports were 0.88 and 0.89 for emotional problems and 0.92 and 0.96 for behavioural problems, respectively.

##### P‐factor at age 18

We focussed on the p‐factor at age 18 because it reflects general liability to a range of psychiatric disorders associated with ACEs (Schaefer et al., 2018). E‐Risk members were assessed in private interviews about past‐year symptoms of mental disorders, including alcohol dependence, cannabis dependence, conduct disorder, tobacco dependence, attention deficit/hyperactivity disorder, depression, generalised anxiety disorder, eating disorders, post‐traumatic stress disorder, psychotic experiences and prodromal symptoms. The Diagnostic Interview Schedule (Robins, Cottler, Bucholz, & Compton, 1995) was used to assess symptoms of alcohol dependence, cannabis dependence, depression, generalised anxiety disorder and post‐traumatic stress disorder. Conduct disorder was measured by inquiring about DSM–IV symptoms (American Psychiatric Association, 1994); symptoms of tobacco dependence were assessed with the Fagerstrom Test for Nicotine Dependence (Heatherton, Kozlowski, Frecker, & Fagerström, 1991) and attention deficit/ hyperactivity disorder was measured by inquiring about Diagnostic and Statistical Manual of Mental Disorders (American Psychiatric Association, 2013) symptoms (Agnew‐Blais et al., 2016). Symptoms of eating disorder were assessed with the SCOFF (Morgan, Reid, & Lacey, 1999). We assessed symptoms of thought disorder in two ways: First, each E‐Risk member was interviewed about delusions and hallucinations (e.g. ‘Have other people ever read your thoughts?’; ‘Have you ever thought you were being followed or spied on?’; ‘Have you ever heard voices that other people cannot hear?’). This interview was also administered at an earlier age to E‐Risk members and its scoring system is described in detail elsewhere (Polanczyk et al., 2010). Second, each E‐Risk member was asked about unusual thoughts and feelings (e.g. ‘My thinking is unusual or frightening’; ‘People or places I know seem different’), drawing on item pools since formalised in prodromal psychosis instruments, including the PRIME‐screen and SIPS (Loewy, Pearson, Vinogradov, Bearden, & Cannon, 2011). The p‐factor is a latent measure of general psychopathology, derived from confirmatory factor analysis based on the 11 observed mental health variables assessed in the interviews (Schaefer et al., 2018).

#### Protective factors

##### Maternal warmth

We assessed maternal warmth at ages 5 and 10 using procedures adapted from the Five Minute Speech Sample method (Magaña et al., 1986). Mothers were asked to speak for 5 minutes about each of their children when they were aged 5 and again at age 10 (Caspi et al., 2004). Warmth is a global measure of the whole speech sample and was assessed by two trained raters for the tone of voice, spontaneity, sympathy and/or empathy towards the child. Warmth was coded on a 6‐point scale. High warmth (5) and moderately high warmth (4) were coded when there was definite warmth, enthusiasm, interest in and enjoyment of the child. Moderate warmth (3) was coded when there was definite understanding, sympathy and concern but only limited warmth of tone. Some warmth (2) was coded when there was a detached and rather clinical approach, with little or no warmth of tone, but moderate understanding, sympathy and concern. Very little warmth (1) was rated when there was only a slight amount of understanding, sympathy or concern or enthusiasm about or interest in the child. No warmth (0) was reserved for respondents who showed a complete absence of the qualities of warmth as defined. Two trained raters coded the tapes of the mothers' speech sample. Inter‐rater reliability was established by having the raters individually code audiotapes describing 40 children. The inter‐rater agreement for maternal warmth was *r* = .90 (Caspi et al., 2004). The raters were blind to all other E‐Risk study data. Scores for maternal warmth at age 5 (*M* = 3.36, *SD* = 0.98) were significantly associated with scores at age 10 (*M* = 3.73, *SD* = 0.89; *r* = .38, *p* < .01) (Bowes et al., 2010). The measures of maternal warmth were therefore averaged across the two time‐points to create a single score.

##### Adult support

At age 12, children answered 13 items assessing the presence of a supportive adult, such as, ‘There is an adult who is looking out for me’, ‘There is an adult who I can tell almost anything to’ and ‘There is an adult who I trust’. Children rated each item as being ‘not true’, ‘sometimes true’ or ‘true’. We coded 0 = ‘not true’, 1 = ‘sometimes true’ and 2 = ‘true’ and summed the responses to create a total score. The internal consistency across the 13 items was α = 0.85.

#### Prior mental health

As covariates, we included baseline emotional and behavioural problems. These were assessed at age 5 using the Child Behavior Checklist with mothers (Achenbach, 1991b) and the Teachers' Report Form with teachers (Achenbach, 1991c), similar to the assessments at age 12. The behavioural problems scale is the sum of items from the delinquency and aggression scales, whilst the emotional problems scale is the sum of items from the withdrawn and anxious/depressed scales, excluding somatic complaints.

### Statistical analysis

Analyses were pre‐registered on the Open Science Framework (https://osf.io/e82bs) and code is publicly available (https://github.com/sestock/adult‐warmth‐and‐support). Analyses were conducted in R Version 4.1.1.

First, we examined the relationship between ACEs experienced between birth and age 12 and three mental health outcomes: (a) emotional problems at age 12, (b) behavioural problems at age 12 and (c) the p‐factor at age 18. This allowed us to identify the minimum ACE score which was associated with elevated risk of mental health problems, so that we could examine protective factors in this at‐risk group. To do so, we used generalised estimating equation (GEE) linear regression models with an exchangeable correlation structure to account for familial clustering. The reference category was children with no ACEs. In exploratory analysis, we used GEE linear regressions to test for interactions between (a) exposure to ACEs and each protective factor and (b) sex and each protective factor in predicting mental health outcomes.

Second, we examined the protective effects of warm and supportive adult involvement on mental health problems, among children exposed to ACEs. To test this, we used GEE linear regressions accounting for familial clustering, with six separate models pairing each protective factor (maternal warmth, adult support) with each mental health outcome (emotional problems at age 12, behavioural problems at age 12 and the p‐factor at age 18). These analyses estimate unadjusted ‘phenotypic’ effects that do not account for familial confounding. ACE exposure was defined as the minimum ACE score that was associated with higher levels of mental health problems in the initial analysis to maximise sample size.

Third, we examined whether the presence of a warm and supportive adult has an environmentally driven protective effect on mental health problems, in the context of ACEs. To do so, we used mixed‐effects twin‐differences GEEs, which estimate *within‐twin pair* and *between‐twin pair* effects (Carlin, Gurrin, Sterne, Morley, & Dwyer, 2005). The within‐twin pair effects show whether a twin who experienced greater levels of maternal warmth and adult support has fewer mental health problems than their co‐twin, thereby accounting for confounding from the shared environment and genetic influences. We focussed first on both monozygotic (MZ) and dizygotic (DZ) twins, before repeating the analysis on MZ twins only to completely rule out genetic confounding. Analyses were restricted to twin pairs with ACE exposure. Notably, ACE scores did not differ between twins in a family (within‐twin pair *r* for MZ and DZ twins' ACE scores = 0.99), because most ACEs are family‐level experiences (e.g. parental separation, parental antisocial behaviour and family history of mental health problems).

Fourth, to minimise confounding from the non‐shared environment, we further adjusted the twin‐differences models for earlier mental health problems at age 5. For analyses focusing on emotional problems at age 12, we controlled for within‐twin differences in emotional difficulties at age 5; for behavioural problems at age 12, we controlled for within‐twin differences in behavioural difficulties at age 5; for the p‐factor at age 18, we controlled for within‐twin differences in both emotional and behavioural difficulties at age 5.

## Results

### Are ACEs associated with mental health problems in childhood and adolescence?

Descriptive statistics for mental health outcomes and protective factors according to ACE exposure are shown in Table 1. Children who experienced one or more ACEs had higher levels of emotional problems at age 12 (β = 0.26; 95% CI = 0.16 to 0.36), behavioural problems at age 12 (β = 0.46, 95% CI = 0.36 to 0.56) and p‐factor scores at age 18 (β = 0.34, 95% CI = 0.23 to 0.44), compared to children who had experienced no ACEs. Therefore, we focussed our further analyses on examining whether warm and supportive adult involvement protects against mental health problems among children exposed to one or more ACEs.

**Table 1:** Descriptive statistics for mental health outcomes and protective factors according to ACE exposure

	No ACE exposure			One or more ACEs		
	*N* (%)	Mean	*SD*	*N* (%)	Mean	*SD*
Mental health outcomes						
Emotional problems at age 12	698 (32.7)	9.55	7.27	1,439 (67.3)	11.66	8.68
Behavioural problems at age 12	698 (32.6)	11.24	11.23	1,440 (67.4)	17.78	15.44
P‐Factor at age 18	659 (32.8)	96.49	13.89	1,350 (67.2)	101.58	15.20
Protective factors						
Maternal warmth at ages 5 and 10	696 (32.7)	3.57	0.79	1,434 (67.3)	3.43	0.84
Adult support at age 12	688 (32.6)	24.27	3.01	1,422 (67.4)	23.53	3.61

*N* indicates total individuals. p‐factor scores were scaled to a mean of 100 and standard deviation of 15.

ACE, adverse childhood experiences; p‐Factor, latent variable of general psychopathology; *SD*, standard deviation.

### Is warm and supportive adult involvement associated with fewer mental health problems in children exposed to adversity?

**Figure 1:**
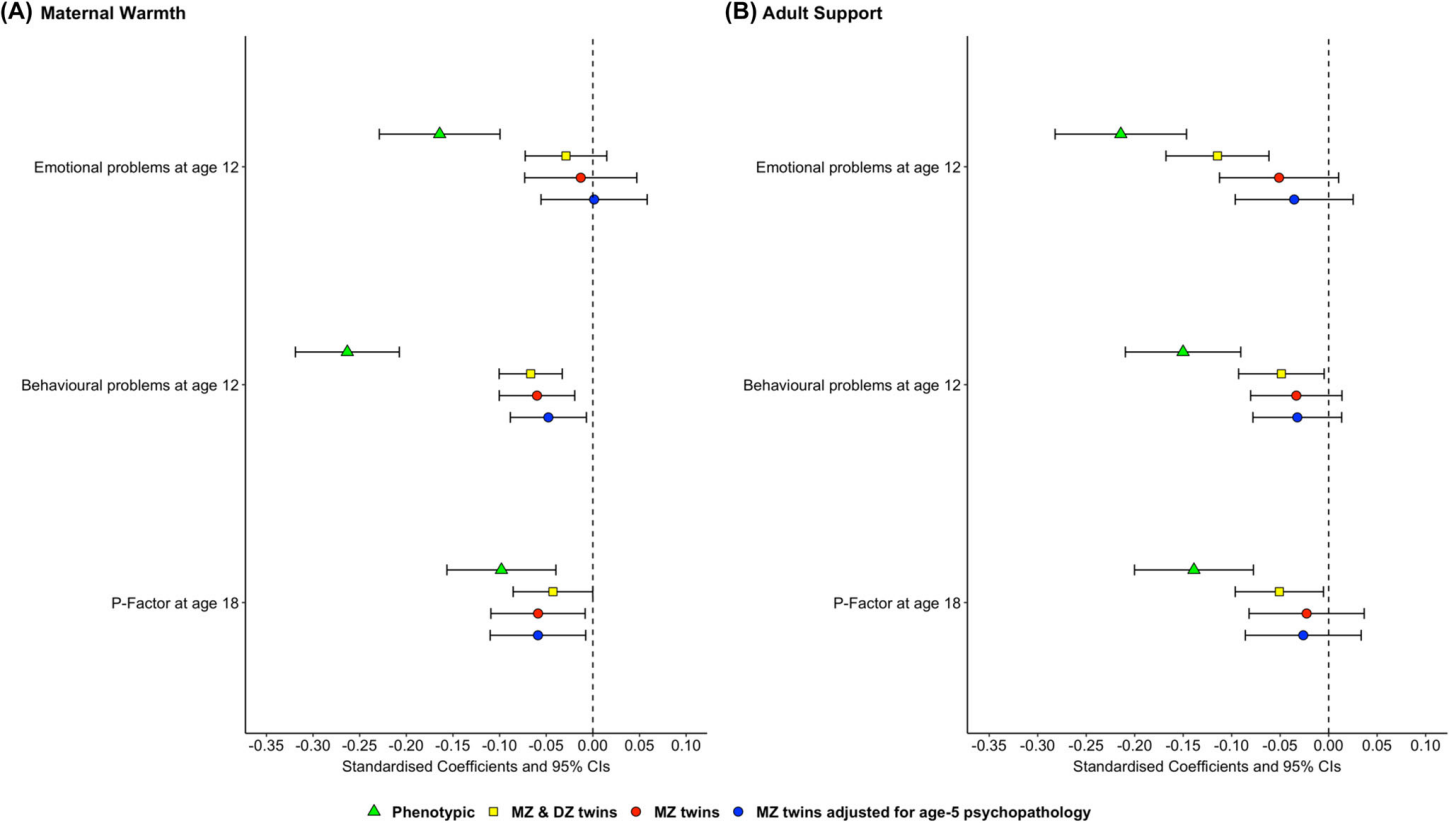
Phenotypic and twin‐difference associations between warm and supportive adult involvement and mental health problems, amongst children exposed to ACEs. Panel A shows the associations between maternal warmth and mental health problems; Panel B shows the associations between adult support and mental health problems. ACEs, adverse childhood experiences; DZ, dizygotic twins; MZ, monozygotic twins; p‐Factor, latent variable of general psychopathology

Phenotypic analyses suggested a protective effect of maternal warmth and adult support on mental health outcomes following ACEs. Among children exposed to ACEs, those who experienced greater maternal warmth had lower levels of emotional and behavioural difficulties at age 12 and lower p‐factor scores at age 18 (Table 2, Panel A; Figure 1A [green triangles]). Similarly, in the context of ACEs, children who experienced greater adult involvement had lower levels of emotional and behavioural difficulties at age 12 and lower p‐factor levels at age 18 (Table 2, Panel A; Figure 1B [green triangles]). Exploratory analyses did not find consistent interaction effects between ACEs and maternal warmth/adult support (Table S2), suggesting that warm and supportive adult involvement may be protective against mental health problems for all children (indicating a ‘promotive’ effect; Brumley & Jaffee, 2016). Furthermore, we did not find interactions between sex and maternal warmth or adult support (Table S3).

**Table 2:** Associations between warm and supportive adult involvement and mental health problems, among children exposed to ACEs

	Panel A phenotypic association	Panel B MZ and DZ twin pairs	Panel C MZ twin pairs	Panel D MZ twin pairs (adjusted for age‐5 psychopathology)
Maternal warmth				
Emotional problems (age 12)	−0.16 (−0.23 to −0.10)	−0.03 (−0.07 to 0.02)	−0.01 (−0.07 to 0.05)	0.001 (−0.06 to 0.06)
Behavioural problems (age 12)	−0.26 (−0.32 to −0.21)	−0.07 (−0.10 to −0.03)	−0.06 (−0.10 to −0.02)	−0.05 (−0.09 to −0.01)
P‐factor (age 18)	−0.10 (−0.16 to −0.04)	−0.04 (−0.09 to −0.0001)	−0.06 (−0.11 to −0.01)	−0.06 (−0.11 to −0.01)
Adult support				
Emotional problems (age 12)	−0.21 (−0.28 to −0.15)	−0.11 (−0.17 to −0.06)	−0.05 (−0.11 to 0.01)	−0.04 (−0.10 to 0.03)
Behavioural problems (age 12)	−0.15 (−0.21 to −0.09)	−0.05 (−0.09 to −0.005)	−0.03 (−0.08 to 0.01)	−0.03 (−0.08 to 0.01)
P‐factor (age 18)	−0.14 (−0.20 to −0.08)	−0.05 (−0.10 to −0.01)	−0.02 (−0.08 to 0.04)	−0.03 (−0.09 to 0.03)

Phenotypic associations are associations between protective factors and mental health outcomes with each twin treated as an individual, accounting for familial clustering. Other columns show associations between within‐twin pair differences.

Results are standardised regression coefficients with 95% confidence intervals in brackets. The number of twin pairs ranged from 683 to 719 for phenotypic analyses; 672 to 719 for MZ and DZ twin pairs and 374 to 395 for MZ twin pairs only. Results from DZ twin pairs can be found in Table S4.

ACEs, adverse childhood experiences; DZ, dizygotic twins; MZ, monozygotic twins; p‐Factor, latent variable of general psychopathology.

### Is warm and supportive adult involvement associated with fewer mental health problems, independent of familial confounding?

We next used the twin‐difference design to test whether the negative associations between warm and supportive adult involvement and mental health problems following ACEs were independent of familial confounding. There was a reasonable amount of within‐twin pair variation in maternal warmth, supportive adult involvement and mental health outcomes at ages 12 and 18 (Table 3; Figure S2), suggesting these variables were suitable for twin‐difference analyses.

**Table 3:** Within‐twin pair correlations for protective factors and outcomes, among twins exposed to 1+ ACEs

	MZ twins	DZ twins
Mental health outcomes		
Emotional problems at age 12	0.47	0.21
Behavioural problems at age 12	0.71	0.47
P‐Factor at age 18	0.51	0.29
Protective factors		
Maternal warmth at ages 5 and 10	0.70	0.61
Adult support at age 12	0.37	0.22

The number of twin pairs ranged from 374 to 395 for MZ twins and 298 to 324 for DZ twins.

ACEs, adverse childhood experiences; DZ, dizygotic twins; MZ, monozygotic twins; p‐Factor, latent variable of general psychopathology.

Among children exposed to ACEs, twins who experienced more maternal warmth had slightly less behavioural difficulties at age 12 and lower p‐factor scores at age 18 compared to their co‐twins (Figure 1A, yellow squares; Table 2, Panel B). However, maternal warmth was not significantly associated with emotional problems at age 12 in twin‐difference analyses (Figure 1A [yellow squares]; Table 2, Panel B), and effect sizes for all outcomes were substantially attenuated (by 60–81%, average decrease = 71%) relative to the phenotypic associations. When restricting the analysis to genetically identical MZ twins (Figure 1A, red circles; Table 2, Panel C), effect sizes were broadly similar to twin‐difference estimates for both MZ & DZ twins, with an average attenuation of 70% from phenotypic associations.

Among children exposed to ACEs, twins with more adult support had slightly fewer mental health problems at ages 12 and 18 than their co‐twins (Figure 1B, yellow squares; Table 2, Panel B), but effect sizes were attenuated by 48%–67% (average decrease = 60%) relative to the phenotypic associations. When restricting to genetically identical MZ twins, associations were attenuated further (Figure 1B, red circles; Table 2, Panel C), with effect sizes decreased by 76%–86% (average decrease = 81%) compared to phenotypic analyses.

When controlling for twin differences in psychopathology at age 5, effect sizes remained similar, with null or minimal associations between adult warmth and support and mental health (Figure 1, blue circles; Table 2, Panel D).

## Discussion

In this pre‐registered, genetically informed longitudinal study, we examined the protective role of maternal warmth and adult support against mental health problems in children exposed to ACEs. In line with previous phenotypic research (Fritz et al., 2018; Sciaraffa et al., 2018), we found that children exposed to ACEs who received greater maternal warmth and adult support had fewer mental health problems in adolescence. However, twin‐difference analyses showed that the protective effects of maternal warmth and adult support on mental health outcomes for children exposed to ACEs were largely accounted for by pre‐existing genetic and environmental factors.

Our findings challenge the assumption that maternal warmth and adult support could protect children exposed to ACEs from developing mental health problems. Instead, they suggest that in the context of ACEs, children with pre‐existing familial risk factors may be less likely to experience maternal warmth and adult support, and more likely to develop psychopathology—resulting in the phenotypic associations. Indeed, after accounting for such familial vulnerabilities in MZ twin‐difference analyses, effect sizes were attenuated from phenotypic analyses on average by 70%–81% (for maternal warmth and adult support, respectively). Our results highlight the importance of genetic vulnerabilities and the family environment (e.g. socioeconomic position) in explaining the associations between adult warmth and support with mental health, following childhood adversity.

Our findings are partly consistent with a previous twin‐difference study in this cohort, which found that maternal warmth was not associated with emotional adjustment in children exposed to bullying, though unique environmental effects on behavioural adjustment were observed (Bowes et al., 2010). Similar to this study, we also found greater attenuation for the effects of maternal warmth on emotional problems relative to behavioural problems in twin‐difference analyses. This suggests that there may be greater overlap between familial factors (e.g. genetic predispositions) that influence maternal warmth and child emotionality, relative to child behaviour problems. However, in contrast to the Bowes et al. (2010) study, we found only a minimal association between maternal warmth and behavioural problems in twin‐difference analyses. This slight difference in findings suggests that maternal warmth may protect against behavioural problems following adversity outside of the home (i.e. for bullying), but not when adversity happens in the home (i.e. for ACEs in the family, as in this study).

It is also important to emphasise that these findings do not mean that social support more generally does not causally protect against mental health problems. While we found minimal or no causal effects of maternal warmth and adult support in children exposed to ACEs, it is possible that other forms of social support (e.g. from peers) may be protective against mental health problems following adversities. Indeed, a previous twin‐difference study in this cohort found that social support from friends was protective against psychotic experiences among poly‐victimised adolescents, independent of familial confounding (Crush et al., 2020). It is also likely that social support has a protective effect against mental health problems in adulthood. Longitudinal studies using fixed‐effects analysis have identified direct effects of social support on adult mental health outcomes (Feng & Astell‐Burt, 2016; Milner, Krnjacki, Butterworth, & LaMontagne, 2016). Finally, it is possible that maternal warmth and adult support are causally protective against mental health problems among certain subpopulations of individuals exposed to ACEs, but not at the average population level. Future research could assess whether different subgroups of children exposed to varying adversity profiles may benefit differently from maternal warmth and adult support.

This study has a number of limitations which should be considered when interpreting the findings. First, adult support was measured by childrens' self‐reports at a single time‐point, and thus could be influenced by mood or concurrent mental health symptoms, which are heritable. Therefore, the genetic confounding observed for the associations between adult support and child mental health may partly reflect genetically influenced perception bias (Pingault et al., 2022), as children with genetic liability to mental health problems may perceive a lack of adult support. Notably though, we accounted for childrens' pre‐existing mental health problems in the analysis. Furthermore, the effects of adult support were similar to those for maternal warmth which was assessed through independently rated maternal speech samples at two time‐points, and therefore is unlikely to be influenced by perception bias. Second, the use of difference scores in the twin differences design can induce measurement error in the protective factors measures and in turn can attenuate co‐twin estimates even in the absence of familial confounding (Frisell, Öberg, Kuja‐Halkola, & Sjölander, 2012; McGue, Osler, & Christensen, 2010). Therefore, it will be important to examine whether these findings triangulate across other quasi‐experimental and genetically informative designs, with different assumptions. Finally, it is possible that findings are not generalisable to singletons, given the use of a twin cohort. However, the prevalence of ACEs in E‐Risk is similar to a non‐twin sample from another UK cohort (Houtepen, Heron, Suderman, Tilling, & Howe, 2018), and levels of psychopathology are generally comparable between twins and individuals (Nøvik, 1995).

To conclude, our findings suggest that maternal warmth and adult social support have minimal protective effects against mental health problems following ACEs. Instead, the observed association between a warm and supportive adult presence with resilience to mental health problems appears to reflect confounding by pre‐existing genetic and environmental influences. These findings have implications for future research and interventions. Regarding future research, our study highlights the value of using a genetically informed design to strengthen causal inference about the role of protective factors in promoting resilience after adversity. Future studies using genetically informed designs and other causal inference methods are needed to strengthen evidence about the effects of other putative protective factors in promoting resilience.

Regarding interventions, our study suggests that practitioners should not assume that programmes aiming to boost maternal warmth and adult support will easily prevent children exposed to adversity from developing mental health problems. The literature demonstrating the phenotypic associations without considering the role of genetic confounding and the family environment has encouraged over‐optimistic expectations of adult social support interventions. Our study suggests that interventions targeted towards improving maternal warmth and adult support would likely also need to address wider family environmental risks and heritable vulnerabilities to substantially boost mental health in adolescents exposed to ACEs.

## Ethical approval

The Joint South London and Maudsley and the Institute of Psychiatry Research Ethics Committee approved each phase of the study. Parents gave informed consent and twins gave assent at 5 to 12 years of age and then informed consent at 18 years.

**Key points**

- Maternal warmth and adult support are associated with less mental health problems following childhood adversity, but it is unclear whether these associations are causal. We aimed to approach causal inference using the twin‐difference design.
- We found that among children exposed to adversity, those who experienced greater maternal warmth and adult support had lower levels of mental health problems across adolescence.
- However, twins who experienced greater maternal warmth and adult support had minimal differences in mental health compared to their co‐twins concordant for adversity exposure, suggesting familial confounding.
- Our findings suggest that interventions which boost maternal warmth and adult support will need to also address wider family environments and heritable vulnerabilities in children exposed to adversity, to improve adolescent mental health.

## Supporting information


**Appendix S1.** Measurement of adverse childhood experiences (ACEs).
**Table S1**. Description of ACE variable measurement.
**Table S2**. Adjusted estimates of interaction effects between ACEs and protective factors with mental health problems.
**Table S3**. Estimates for interaction effects between sex and protective factors with mental health problems.
**Table S4**. Associations between warm and supportive adult involvement and mental health problems, amongst DZ twins exposed to ACEs.
**Figure S1**. Distribution of E‐Risk participants at age 18 across index of multiple deprivation deciles.
**Figure S2**. Histograms of within‐twin differences in mental health outcomes and protective factors.

## Data Availability

The data that support the findings of this study are not publicly available but can be accessed with permission from the E‐Risk Study team: https://eriskstudy.com/data‐access/.
