# Supplementary material for: Can a warm and supportive adult protect against mental health problems amongst children with experience of adversity? A twin‐differences study
Source: J Child Psychol Psychiatry. 2024 Nov 12;66(5):650–8. doi: 10.1111/jcpp.14070 (PMC12018152; doi:10.1111/jcpp.14070)
Supplement: Supplementary file 1 — Appendix S1. Measurement of adverse childhood experiences (ACEs). Table S1. Description of ACE variable measurement. Table S2. Adjusted estimates of interaction effects between ACEs and protective factors with mental health problems. Table S3. Estimates for interaction effects between sex and protective factors with mental health problems. Table S4. Associations between warm and supportive adult involvement and mental health problems, amongst DZ twins exposed to ACEs. Figure S1. Distribution of E‐Risk participants at age 18 across index of multiple deprivation deciles. Figure S2. Histograms of within‐twin differences in mental health outcomes and protective factors. [file JCPP-66-650-s001.docx]

Can a warm and supportive adult protect against mental health problems amongst children with experience of adversity? A twin-differences study

Supporting Information

Table of Contents

[Appendix S1. Measurement of adverse childhood experiences (ACEs) 2](#_Toc179875326)

[Table S1. Description of ACE variable measurement 3](#_Toc179875327)

[Table S2. Adjusted estimates of interaction effects between ACEs and protective factors with mental health problems 5](#_Toc179875328)

[Table S3. Estimates for interaction effects between sex and protective factors with mental health problems 5](#_Toc179875329)

[Table S4. Associations between warm and supportive adult involvement and mental health problems, amongst DZ twins exposed to ACEs 6](#_Toc179875330)

[Figure S1. Distribution of E-Risk participants at age 18 across Index of Multiple Deprivation deciles 6](#_Toc179875331)

[Figure S2. Histograms of within-twin differences in mental health outcomes and protective factors 8](#_Toc179875332)

[Supporting Information References 9](#_Toc179875333)

Appendix S1. Measurement of adverse childhood experiences (ACEs)

The E-Risk Study assessed five types of child harm (physical abuse, sexual abuse, emotional abuse, emotional neglect, and physical neglect) and five types of household dysfunction (household partner violence, household substance abuse, family mental illness, parental antisocial behaviour, and parental separation) to correspond to the 10 categories of childhood adversity introduced by the CDC Adverse Childhood Experiences Study.^1^ These experiences were assessed from records gathered over four home visits from ages 5 to 12 years. These records included the following: structured notes from interviews with parents using the MultiSite Child Development Project interview^2,3^ (to assess child harm), the Conflict Tactics Scale^4^ (to assess household partner violence), the Young Adult Behavior Checklist^5^ (to assess parental antisocial behaviour), and the Family History Screen^6,7^ (to assess household substance abuse and family history of mental illness); observations by research workers of the family environment using the Home Observation for Measurement of the Environment^8^ (including assessments of parent-child interactions and whether the environment was safe, sanitary and healthy), and information from clinicians whenever the E-Risk Study team made a child-protection referral.

Each category of childhood adversity was coded as a binary exposure: physical abuse, emotional abuse, physical neglect, emotional neglect, and sexual abuse were coded if there was evidence of severe exposure;^9^ household partner violence was coded if there was evidence that it was repeated; household substance abuse was defined as approximately the top quarter of the proportion of family members with a history of substance use; family mental illness was defined as a report of hospitalisation for psychiatric disorder or substance-use problem, or attempted or completed suicide for any of the child’s biological mother, father, grandparents, or aunts and uncles; parental antisocial behaviour was coded as the top quarter of the variety of antisocial behaviours of parents, and parental separation was defined as one or both biological parent(s) being absent from the household at some point. Further descriptions of each component ACE measure can be found in Table S1.

ACE summary score. We derived an ACE summary score by summing the dichotomised component measures (namely, physical abuse, sexual abuse, emotional abuse and neglect, physical neglect, household partner violence, household substance abuse, family mental illness, parental antisocial behaviour, and parental separation).

Table S1. Description of ACE variable measurement

| **ACEs** | **Age** | **Source** | **Prevalence n (%)** | **Description** |
| --- | --- | --- | --- | --- |
| Physical abuse | Birth-12 | Life dossier | 114 (5.1%) | Dichotomous indicators of severe forms of maltreatment. The study developed a cumulative profile for each child across ages 5, 7, 10, and 12. The profile comprised the caregiver reports of maltreatment, recorded debriefings with interviewers who had coded any indication of maltreatment at any of the successive home visits, recorded narratives of the successive caregiver interviews, and information from clinicians whenever the study team made a child-protection referral. The profiles were reviewed at the end of the age 12 phase by two clinical psychologists. Initial inter-rater agreement between the coders was 90% of cases for whom maltreatment was identified (100% for cases of sexual abuse), and discrepantly coded cases were resolved by consensus review (For additional details see Danese et al. 2016). |
| Sexual abuse | Birth-12 | Life dossier | 16 (0.7%) |  |
| Emotional abuse or neglect | Birth-12 | Life dossier | 68 (3.1%) |  |
| Physical neglect | Birth-12 | Life dossier | 44 (2.0%) | Dichotomous indicator of severe physical neglect defined as any sign that the caretaker was not providing a safe, sanitary, or healthy environment for the child. This included the child not having proper clothing or food, as well as grossly unsanitary home environments. Initial inter-rater agreement between the coders was 85% of cases for whom maltreatment was identified, and discrepantly coded cases were resolved by consensus review (For additional details see Danese et al. 2016). |
| Domestic violence exposure | Birth-10 | Mother-report | 374 (16.8%) | Dichotomous indicator of exposure to severe domestic violence as reported by mothers. Mothers reported about perpetration of and victimisation involving 12 forms of physical violence (e.g., slapping, hitting, kicking, and strangling) from the Conflict Tactics Scale (Straus 1990), when the children were 5, 7, and 10 years of age). Reports of either perpetration or victimisation constituted evidence of physical domestic violence (For additional details see Danese et al. 2016). |
| Parental antisocial behaviour | 5 | Mother-report | 560 (25.2%) | Dichotomous indicator of approximately the top quarter of the variety of antisocial behaviours of parents. Father’s and mother’s history of antisocial behavior was reported by the mothers when the children were 5 years old. Mothers were interviewed using the Young Adult Behavior Checklist (Achenbach 1997), which was modified to obtain lifetime data. Full details of father’s and mother’s history of antisocial behavior within the E-Risk Study are reported elsewhere (Jaffee et al. 2003). A study of mother–father agreement about men’s antisocial behaviour in this sample showed that women provided reliable information about the behaviour of their children’s father (Caspi et al. 2001). |
| Family history of substance abuse | 12 | Mother-report | 478 (22.3%) | Dichotomous indicator of approximately the top quarter of the proportion of family members with a history of substance use for any of the child’s biological mother, father, maternal grandparents, or maternal aunts and uncles. Family history algorithms follow those outlined by Milne et al. (2008). |
| Family history of mental health disorders | 12 | Mother-report | 658 (31.0%) | Dichotomous indicator of a family history of a report of hospitalisation for psychiatric disorder or attempted or completed suicide for any of the child’s biological mother, father, maternal grandparents, or maternal aunts and uncles (For additional details see Belsky et al. 2012). |
| Parental separation or divorce | Birth-10 | Mother-report | 1024 (48.0%) | Dichotomous indicator of biological parent separation or divorce as assessed at each interview, up to 10 years of age, by questions on whether the biological parents were living in the same household. |

Note. This table is adapted from Beckley et al.^10^. ACEs, adverse childhood experiences.

Table S2. Adjusted estimates of interaction effects between ACEs and protective factors with mental health problems

|  | β | 95% CI |
| --- | --- | --- |
| **Emotional Problems (age 12)** |  |  |
| **Maternal warmth** | -0.18 | -0.26, -0.10 |
| ACEs | 0.23 | 0.14, 0.31 |
| ACEs*Maternal warmth | -0.01 | -0.10, 0.08 |
| **Adult support** | -0.07 | -0.16, 0.01 |
| ACEs | 0.22 | 0.13, 0.31 |
| ACEs*Adult support | -0.15 | -0.24, -0.05 |
| **Behavioural Problems (age 12)** |  |  |
| **Maternal warmth** | -0.16 | -0.23, -0.09 |
| ACEs | 0.42 | 0.33, 0.50 |
| ACEs*Maternal warmth | -0.16 | -0.25, -0.07 |
| **Adult support** | -0.15 | -0.23, -0.07 |
| ACEs | 0.41 | 0.32, 0.50 |
| ACEs*Adult support | -0.07 | -0.16, 0.03 |
| **P-Factor (age 18)** |  |  |
| **Maternal warmth** | -0.04 | -0.12, 0.04 |
| ACEs | 0.33 | 0.24, 0.42 |
| ACEs*Maternal warmth | -0.06 | -0.16, 0.03 |
| **Adult support** | -0.12 | -0.20, -0.03 |
| ACEs | 0.31 | 0.22, 0.40 |
| ACEs*Adult support | -0.04 | -0.14, 0.06 |
| Note: ACEs, adverse childhood experiences. CI= Confidence Intervals. ACEs are coded to represent no ACEs (0) or one or more ACEs (1). | | |

Table S3. Estimates for interaction effects between sex and protective factors with mental health problems

|  | β | 95% CI |
| --- | --- | --- |
| **Emotional Problems (age 12)** |  |  |
| **Maternal warmth** |  |  |
| Sex*Maternal warmth | 0.04 | -0.09, 0.17 |
| **Adult support** |  |  |
| Sex*Adult support | 0.01 | -0.14, 0.15 |
| **Behavioural Problems (age 12)** |  |  |
| **Maternal warmth** |  |  |
| Sex*Maternal warmth | 0.09 | -0.02, 0.20 |
| **Adult support** |  |  |
| Sex*Adult support | -0.004 | -0.11, 0.10 |
| **P-Factor (age 18)** |  |  |
| **Maternal warmth** |  |  |
| Sex*Maternal warmth | -0.05 | -0.17, 0.07 |
| **Adult support** |  |  |
| Sex*Adult support | -0.09 | -0.21, 0.04 |
| Note: ACEs, adverse childhood experiences. CI= Confidence Intervals. Sex is coded to represent male (0) or female (1). Analysis is unadjusted. | | |

Table S4. Associations between warm and supportive adult involvement and mental health problems, amongst DZ twins exposed to ACEs

|  | **β** | **95% CIs** |
| --- | --- | --- |
| **Maternal Warmth** |  |  |
| Emotional problems (age 12) | -0.05 | -0.11, 0.02 |
| Behavioural problems (age 12) | -0.07 | -0.13, -0.02 |
| P-factor (age 18) | -0.02 | -0.09, 0.05 |
| **Adult support** |  |  |
| Emotional problems (age 12) | -0.18 | -0.27, -0.10 |
| Behavioural problems (age 12) | -0.06 | -0.14, 0.01 |
| P-factor (age 18) | -0.08 | -0.15, -0.01 |

Note: ACEs, adverse childhood experiences. CIs, confidence intervals. DZ, dizygotic twins. P-Factor, latent variable of general psychopathology.

Figure S1. Distribution of E-Risk participants at age 18 across Index of Multiple Deprivation deciles

| Participants (%) |  |
| --- | --- |
|  | Index of Multiple Deprivation Deciles  (1=most deprived, 10=least deprived) |

Note: This histogram shows E-Risk families’ addresses are a near-perfect match to the deciles of the UK’s 2015 Lower-layer Super Output Area (LSOA) Index of Multiple Deprivation (IMD) which averages 1,500 residents (or 650 households each); approximately 10% of the E-Risk cohort fills each of the IMD’s 10% bands, indicating that the E-Risk cohort accurately represents the distribution of deprivation in the UK.

Figure S2. Histograms of within-twin differences in mental health outcomes and protective factors


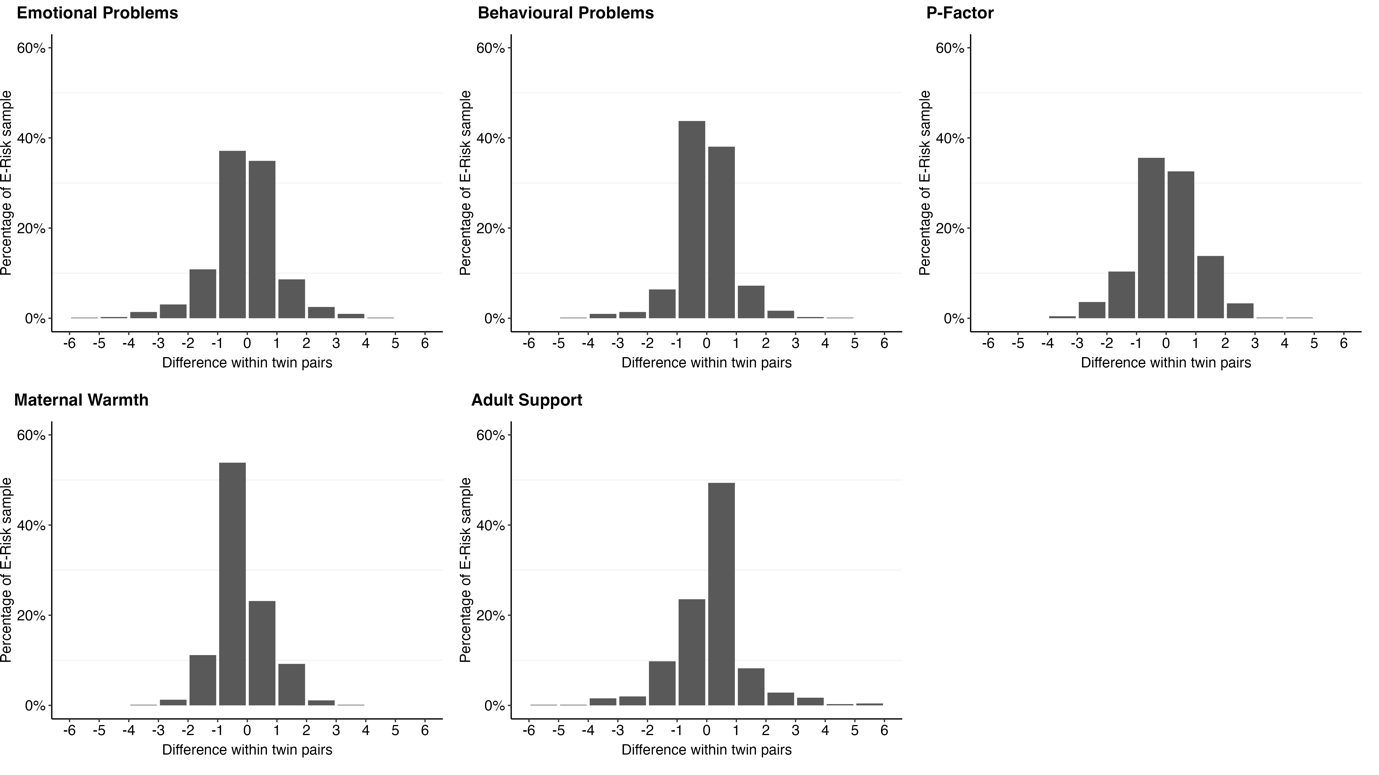


Note: The sample is based on twin pairs who have experienced one or more adverse childhood experience (N=683 to 719). Measures have been standardised to mean=0, SD=1. Difference within twin pairs is the difference between eldest and youngest twin.

Supporting Information References

1. Felitti VJ, Anda RF, Nordenberg D, et al. Relationship of childhood abuse and household dysfunction to many of the leading causes of death in adults: The adverse childhood experiences (ACE) study. *American Journal of Preventive Medicine*. 1998;14(4):245-258. doi:10.1016/S0749-3797(98)00017-8

2. Dodge KA, Bates JE, Pettit GS. Mechanisms in the cycle of violence. *Science*. 1990;250(4988):1678.

3. Lansford JE, Dodge KA, Pettit GS, Bates JE, Crozier J, Kaplow J. A 12-Year prospective study of the long-term effects of early child physical maltreatment on psychological, behavioral, and academic problems in adolescence. *Archives of Pediatrics & Adolescent Medicine*. 2002;156(8):824-830. doi:10.1001/archpedi.156.8.824

4. Straus MA. Measuring intrafamily conflict and violence: The Conflict Tactics (CT) Scales. *Journal of Marriage and Family*. 1979;41(1):75-88. doi:10.2307/351733

5. Achenbach TM. *Manual for the Young Adult Self-Report and Young Adult Behavior Checklist.* University of Vermont, Department of Psychiatry; 1997.

6. Weissman MM, Wickramaratne P, Adams P, Wolk S, Verdeli H, Olfson M. Brief screening for family psychiatric history: The family history screen. *Archives of General Psychiatry*. 2000;57(7):675-682. doi:10.1001/archpsyc.57.7.675

7. Milne BJ, Caspi A, Harrington H, Poulton R, Rutter M, Moffitt TE. Predictive value of family history on severity of illness: The case for depression, anxiety, alcohol dependence, and drug dependence. *Archives of General Psychiatry*. 2009;66(7):738-747. doi:10.1001/archgenpsychiatry.2009.55

8. Bradley RH, Caldwell BM. Home observation for measurement of the environment: a validation study of screening efficiency. *American Journal of Mental Deficiency*. 1977;81(5):417-420.

9. Danese A, Moffitt TE, Arseneault L, et al. The origins of cognitive deficits in victimized children: Implications for neuroscientists and clinicians. *American Journal of Psychiatry*. 2017;174(4):349-361. doi:10.1176/appi.ajp.2016.16030333

10. Beckley AL, Caspi A, Arseneault L, et al. The developmental nature of the victim-offender overlap. *Journal of Developmental and Life-Course Criminology*. 2018;4(1):24-49. doi:10.1007/s40865-017-0068-3
